# Supplementary material for: Design and evaluation of low-cost, DIY programmable tissue processor for solvent exchange in biological sample preparation
Source: PLoS One. 2026 Mar 3;21(3):e0341033. doi: 10.1371/journal.pone.0341033 (PMC12956129; doi:10.1371/journal.pone.0341033)
Supplement: S1 Data — The CAD files, provided in both STEP and SLDPRT formats, are available in the GitHub repository: https://doi.org/10.5281/zenodo.17050743. (DOCX) [file pone.0341033.s001.docx]

**Supporting information**

**Design and evaluation of low-cost, DIY programmable tissue processor for solvent exchange in biological sample preparation**

May Wang^1,§^, Samantha Pelletier^2,§^, Alexis Ellis^3^, Robert Shepherd^1^, Margaret Frank^2^, Abraham Stroock^2,3,4^, Anand Kumar Mishra^1,5,*^, Vesna Bacheva^2,3,4,*^

^§^These authors contributed equally

^*^Corresponding authors: Anand Mishra ([am2877@cornell.edu](mailto:am2877@cornell.edu)),Vesna Bacheva ([vb333@cornell.edu](mailto:vb333@cornell.edu))

^1^Department of Mechanical and Aerospace Engineering, Cornell University, Ithaca, NY 14850, USA

^2^School of Integrative Plant Science, Cornell University, Ithaca, NY 14853, USA

^3^Smith School of Chemical and Biomolecular Engineering, Cornell University, Ithaca 14853, NY, USA

^4^Kavli Institute at Cornell for Nanoscale Science, Cornell University, Ithaca, NY 14853, USA

^5^Department of Mechanical Materials and Aerospace Engineering, West Virginia University, Morgantown, WV 26505, USA

**Software code used to operate the tissue processor**

// Last Updated 7/8/24

// ----- Libraries -----

#include <SPI.h>

#include "Adafruit_GFX.h"

#include "Adafruit_ST7735.h"

#include <Servo.h>

#include "Adafruit_Keypad.h"

#include <Arduino.h>

#include <EEPROM.h>

#include <SD.h>

#include <ArduinoJson.h>

// ----- Calibration Variables -----

// Time for one pump to fill up the 250ml top funnel in ms, default 42000

long timeToFill;

// Time for the top seperatory funnel to drain in ms, default 5000

int timeTopDrain;

// Time for the bottom glass compartment to drain in ms, default 5000

int timeBotDrain;

// Time for the pipe valve to completely open / close in ms, default 5000

int timePipeSwitch;

// Time for the tubes to completely drain in ms, default 12000

int timeTubeDrain;

// ----- Hardware Setup -----

// Screen Setup

#define TFT_CS 10

#define TFT_DC 8

#define SD_CS 11

#define TFT_RST -1

Adafruit_ST7735 tft = Adafruit_ST7735(TFT_CS, TFT_DC, TFT_RST);

// Servo Setup

#define servo 41

#define servoOpen 90

#define servoClosed 180

Servo topServo;

// Pipe Setup

#define pipeOpen 6

#define pipeClose 5

// Pump Setup

#define waterPump 37

#define EtOH95Pump 35

#define EtOH100Pump 33

// Keypad Setup

const byte ROWS = 4;

const byte COLS = 4;

char keys[ROWS][COLS] = { { '1', '2', '3', 'A' }, { '4', '5', '6', 'B' }, { '7', '8', '9', 'C' }, { '*', '0', '#', 'D' } };

//byte rowPins[ROWS] = { 32, 30, 28, 26 };

//byte colPins[COLS] = { 40, 38, 36, 34 };

byte rowPins[ROWS] = {34, 36, 38, 40 };

byte colPins[COLS] = {26, 28,30, 32 };

Adafruit_Keypad customKeypad = Adafruit_Keypad(makeKeymap(keys), rowPins, colPins, ROWS, COLS);

// ----- Data Classes -----

class RoutineIDs {

public:

  RoutineIDs(int* ids, int count) : ids(ids), count(count) {}

  ~RoutineIDs() { delete[] ids; }

  int* getIds() const { return ids; }

  int getCount() const { return count; }

private:

  int* ids;

  int count;

};

class Routine {

public:

  Routine(int* durations, int* concentrations, int size)

      : durations(durations), concentrations(concentrations), size(size) {}

  ~Routine() {

    delete[] durations;

    delete[] concentrations;

  }

  int* getDurations() const { return durations; }

  int* getConcentrations() const { return concentrations; }

  int getSize() const { return size; }

private:

  int* durations;

  int* concentrations;

  int size;

};

// ----- Function Declarations -----

// Arduino Functions

void setup();

void loop();

// Sreen Functions

void clear();

void errorPrint(String error);

void successPrint(String success);

// Keypad Input Functions

char promptSingle();

int captureNumber(String prompt);

// Hardware Functions

void drainTop();

void drainBot();

void fill(int percent);

void flushTubes(int ms);

// JSON Helpers

File openFile(const char* filename, uint8_t mode);

bool readJsonFromFile(const char* filename, DynamicJsonDocument& doc);

bool writeJsonToFile(const char* filename, DynamicJsonDocument& doc);

// SD Card Functions

void checkSD();

void setCalibration();

RoutineIDs* getRoutineIDs();

Routine* getRoutine(int RoutineID);

void addRoutineToJson(int RoutineID, JsonArray durations, JsonArray concentrations);

void deleteRoutineFromJson(int RoutineID);

// Menuing Functions

void mainMenu();

void modifyRoutines();

void settings();

// Time Helpers

unsigned long calculateRemainingTime(int* durations, int start, int end);

unsigned long calculateTotalTime(int* durations, int size);

// Action Functions

void runRoutine();

void newRoutine();

void viewRoutines();

void viewRoutine(int routineID);

void deleteRoutine();

void flush();

void sanityCheck();

void calibrate();

void viewCalibration();

// ----- Function Definitions -----

// Arduino Functions

void setup() {

  tft.initR(INITR_BLACKTAB);

  tft.setRotation(1);

  tft.fillScreen(ST77XX_BLACK);

  tft.setTextSize(1);

  tft.setTextColor(ST77XX_WHITE);

  customKeypad.begin();

  SD.begin(SD_CS);

  checkSD();

  setCalibration();

  topServo.attach(41);

  topServo.write(servoClosed);

  pinMode(waterPump, OUTPUT);

  pinMode(EtOH95Pump, OUTPUT);

  pinMode(EtOH100Pump, OUTPUT);

  pinMode(pipeOpen, OUTPUT);

  pinMode(pipeClose, OUTPUT);

}

void loop() {

  mainMenu();

}

// Screen Functions

void clear() {

  tft.fillScreen(ST77XX_BLACK);

  tft.setCursor(0, 0);

}

void debugPrint(String debug){

  clear();

  tft.setTextColor(ST77XX_YELLOW);

  tft.println(debug);

  tft.println("press any key to continue");

  tft.setTextColor(ST77XX_WHITE);

  char confirmation = promptSingle();

}

void errorPrint(String error){

  clear();

  tft.setTextColor(ST77XX_RED);

  tft.println(error);

  tft.setTextColor(ST77XX_WHITE);

  delay(2000);

}

void successPrint(String success){

  clear();

  tft.setTextColor(ST77XX_GREEN);

  tft.println(success);

  tft.setTextColor(ST77XX_WHITE);

  delay(2000);

}

// Keypad Input Functions

char promptSingle() {

  while (true) {

    customKeypad.tick();

    if (customKeypad.available()) {

      keypadEvent e = customKeypad.read();

      if (e.bit.EVENT == KEY_JUST_PRESSED) {

        return e.bit.KEY;

      }

    }

  delay(100);

  }

}

int captureNumber(String prompt) {

  clear();

  tft.println(prompt);

  tft.println("Press # to submit and * to clear all");

  String input = "";

  char key = 0;

  while (true) {

    customKeypad.tick();

    if (customKeypad.available()) {

      keypadEvent e = customKeypad.read();

      if (e.bit.EVENT == KEY_JUST_PRESSED) {

        key = e.bit.KEY;

        if (key == '#') {

          break;

        } else if (key == '*') {

          input = "";

          clear();

          tft.println(prompt);

          tft.println("Press # to submit and * to delete last digit");

        } else if (key >= '0' && key <= '9') {

          tft.print(key);

          input += key;

        }

      }

    }

    delay(100);

  }

  return input.toInt();

}

// Hardware Functions

void drainTop(){

  topServo.write(servoOpen);

  delay(timeTopDrain);

  topServo.write(servoClosed);

}

void drainBot(){

  digitalWrite(pipeOpen, HIGH);

  delay(timePipeSwitch);

  digitalWrite(pipeOpen, LOW);

  delay(timeBotDrain);

  digitalWrite(pipeClose, HIGH);

  delay(timePipeSwitch);

  digitalWrite(pipeClose, LOW);

}

void fill(int percent){

  if (percent > 95){

    digitalWrite(waterPump, HIGH);

    digitalWrite(EtOH100Pump, HIGH);

    delay(timeToFill-(percent*timeToFill/100));

    digitalWrite(waterPump, LOW);

    delay(percent*timeToFill/100 - (timeToFill-(percent*timeToFill/100)));

    digitalWrite(EtOH100Pump, LOW);

  }

  else{

    long msEtOH95 = timeToFill * percent / 0.95 / 100;

    long msWater = timeToFill - msEtOH95;

    if (msEtOH95 >= msWater){

      digitalWrite(waterPump, HIGH);

      digitalWrite(EtOH95Pump, HIGH);

      delay(msWater);

      digitalWrite(waterPump, LOW);

      delay(msEtOH95 - msWater);

      digitalWrite(EtOH95Pump, LOW);

    } else {

      digitalWrite(waterPump, HIGH);

      digitalWrite(EtOH95Pump, HIGH);

      delay(msEtOH95);

      digitalWrite(EtOH95Pump, LOW);

      delay(msWater - msEtOH95);

      digitalWrite(waterPump, LOW);

    }

  }

}

// JSON File Format

// {

//   "routines": {

//     "*ID1*": {

//       "duration": [10, 10, 10],

//       "concentration": [0, 50, 100]

//     },

//     "*ID2*": {

//       "duration": [10, 10, 10],

//       "concentration": [0, 50, 100]

//     }

//   },

//   "calibration": {

//     "timeToFill": 42000,

//     "timeTopDrain": 5000,

//     "timeBotDrain": 5000,

//     "timePipeSwitch": 5000,

//     "timeTubeDrain": 12000

//   }

// }

// JSON Helpers

File openFile(const char* filename, uint8_t mode) {

  File file = SD.open(filename, mode);

  if (!file) {

    errorPrint(String(filename) + " not found on SD Card");

  }

  return file;

}

bool readJsonFromFile(const char* filename, DynamicJsonDocument& doc) {

  File file = openFile(filename, FILE_READ);

  if (!file) return false;

  DeserializationError error = deserializeJson(doc, file);

  file.close();

  if (error) {

    errorPrint("Failed to parse JSON: " + String(error.c_str()));

    return false;

  }

  return true;

}

bool writeJsonToFile(const char* filename, DynamicJsonDocument& doc) {

  // First, delete the existing file

  if (SD.exists(filename)) {

    SD.remove(filename);

  }

  // Open the file for writing

  File file = SD.open(filename, FILE_WRITE);

  if (!file) {

    errorPrint("Failed to open file for writing");

    return false;

  }

  // Write the JSON data to the file

  if (serializeJson(doc, file) == 0) {

    errorPrint("Failed to write to file");

    file.close();

    return false;

  }

  // Close the file

  file.close();

  return true;

}

// SD Card Functions

void checkSD() {

  if (!SD.exists("ROUTINES.TXT")) {

    errorPrint("Can not find ROUTINES.TXT on sd card, creating file");

    DynamicJsonDocument doc(4096);

    JsonObject root = doc.to<JsonObject>();

    root.createNestedObject("routines");

// Calibration Variables set 7/8/2024 for Cornell Build V1

    JsonObject calibration = root.createNestedObject("calibration");

    calibration["timeToFill"] = 202000;

    calibration["timeTopDrain"] = 19500;

    calibration["timeBotDrain"] = 12500;

    calibration["timePipeSwitch"] = 5000;

    calibration["timeTubeDrain"] = 7500;

    File file = SD.open("ROUTINES.TXT", FILE_WRITE);

    if (file) {

      serializeJson(doc, file);

      file.close();

      successPrint("ROUTINES.TXT created with default content.");

    } else {

      errorPrint("Could not create ROUTINES.TXT");

    }

  }

}

void setCalibration() {

  // Get JSON from file

  File file = SD.open("ROUTINES.TXT", FILE_READ);

  if (!file) {

    errorPrint("ROUTINES.TXT not found on SD Card");

    return;

  }

  DynamicJsonDocument doc(4096);

  DeserializationError error = deserializeJson(doc, file);

  file.close();

  if (error) {

    errorPrint("Failed to parse JSON: " + String(error.c_str()));

    return;

  }

  // Access the calibration object in the JSON document

  JsonObject calibration = doc["calibration"];

  // Set the calibration variables from the JSON values

  timeToFill = calibration["timeToFill"];

  timeTopDrain = calibration["timeTopDrain"];

  timeBotDrain = calibration["timeBotDrain"];

  timePipeSwitch = calibration["timePipeSwitch"];

  timeTubeDrain = calibration["timeTubeDrain"];

}

// Function to get all Routine IDs as a int array

RoutineIDs* getRoutineIDs() {

  DynamicJsonDocument doc(4096);

  if (!readJsonFromFile("ROUTINES.TXT", doc)) return nullptr;

  JsonObject routines = doc["routines"].as<JsonObject>();

  int routineCount = routines.size();

  int* ids = new int[routineCount];

  int index = 0;

  for (JsonPair p : routines) {

    const char* keyString = p.key().c_str();

    ids[index++] = atoi(keyString);

  }

  return new RoutineIDs(ids, routineCount);

}

Routine* getRoutine(int RoutineID) {

  DynamicJsonDocument doc(4096);

  if (!readJsonFromFile("ROUTINES.TXT", doc)) return nullptr;

  String idStr = String(RoutineID);

  JsonObject routine = doc["routines"][idStr].as<JsonObject>();

  JsonArray durations = routine["duration"].as<JsonArray>();

  JsonArray concentrations = routine["concentration"].as<JsonArray>();

  int size = min(durations.size(), concentrations.size());

  int* durationsArray = new int[size];

  int* concentrationsArray = new int[size];

  for (int i = 0; i < size; ++i) {

    durationsArray[i] = durations[i].as<int>();

    concentrationsArray[i] = concentrations[i].as<int>();

  }

  return new Routine(durationsArray, concentrationsArray, size);

}

void addRoutineToJson(int RoutineID, JsonArray durations, JsonArray concentrations) {

  DynamicJsonDocument doc(4096);

  if (!readJsonFromFile("ROUTINES.TXT", doc)) return;

  String idStr = String(RoutineID);

  JsonObject newRoutine = doc["routines"].createNestedObject(idStr);

  newRoutine["duration"] = durations;

  newRoutine["concentration"] = concentrations;

  if (!writeJsonToFile("ROUTINES.TXT", doc)) {

    errorPrint("Could not write to file");

  }

}

void deleteRoutineFromJson(int RoutineID) {

  DynamicJsonDocument doc(4096);

  if (!readJsonFromFile("ROUTINES.TXT", doc)) return;

  String idStr = String(RoutineID);

  JsonObject routines = doc["routines"];

  routines.remove(idStr);

  if (!writeJsonToFile("ROUTINES.TXT", doc)) {

    errorPrint("Could not write to file");

  }

}

void updateCalibrationJson(long newTimeToFill, int newTimeTopDrain, int newTimeBotDrain, int newTimePipeSwitch, int newTimeTubeDrain) {

  DynamicJsonDocument doc(4096);

  if (!readJsonFromFile("ROUTINES.TXT", doc)) return;

  // Update the calibration values

  JsonObject calibration = doc["calibration"];

  calibration["timeToFill"] = newTimeToFill;

  calibration["timeTopDrain"] = newTimeTopDrain;

  calibration["timeBotDrain"] = newTimeBotDrain;

  calibration["timePipeSwitch"] = newTimePipeSwitch;

  calibration["timeTubeDrain"] = newTimeTubeDrain;

    // Open the file for writing

  if (!writeJsonToFile("ROUTINES.TXT", doc)) {

    errorPrint("Could not write to file");

  }

}

// Menuing Functions

void mainMenu() {

  clear();

  tft.setTextColor(ST77XX_GREEN);

  tft.println("Plant Dehydrator V0.3");

  tft.setTextColor(ST77XX_WHITE);

  tft.print("Made By Shawn Wang ");

  tft.setTextColor(0x5e7e);

  tft.print(":");

  tft.setTextColor(0xf556);

  tft.println("3");

  tft.setTextColor(ST77XX_WHITE);

  tft.println("slw282@cornell.edu");

  tft.println("A. Run Routine");

  tft.println("B. Modify Routines");

  tft.println("C. Flush Tubes");

  tft.println("D. Settings");

  char userSelection = promptSingle();

  switch (userSelection) {

    case '*':

      return;

    case 'A':

      runRoutine();

      return;

    case 'B':

      modifyRoutines();

      return;

    case 'C':

      flush();

      return;

    case 'D':

      settings();

      return;

    default:

      mainMenu();

  }

}

void modifyRoutines(){

  clear();

  tft.println("Modify Routines (* to go back):");

  tft.println("A. New Routine");

  tft.println("B. View Routine");

  tft.println("C. Delete Routine");

  char userSelection = promptSingle();

  switch (userSelection) {

    case '*':

      return;

    case 'A':

      newRoutine();

      return;

    case 'B':

      viewRoutines();

      return;

    case 'C':

      deleteRoutine();

      return;

    default:

      modifyRoutines();

  }

}

void settings(){

  clear();

  tft.println("Settings (* to go back):");

  tft.println("A. Sanity Check");

  tft.println("B. Calibrate");

  tft.println("C. View Cal. Values");

  char userSelection = promptSingle();

  switch (userSelection) {

    case '*':

      return;

    case 'A':

      sanityCheck();

      return;

    case 'B':

      calibrate();

      return;

    case 'C':

      viewCalibration();

      return;

    default:

      settings();

  }

}

// Time Helpers

unsigned long calculateRemainingTime(int* durations, int start, int end) {

  unsigned long remainingTime = 0;

  for (int i = start; i < end; ++i) {

    remainingTime += durations[i];

  }

  return remainingTime;

}

unsigned long calculateTotalTime(int* durations, int size) {

  unsigned long totalTime = 0;

  for (int i = 0; i < size; ++i) {

    totalTime += durations[i];

  }

  return totalTime;

}

// Action Functions

void runRoutine() {

  // Step 1: Show the program selection screen

  RoutineIDs* routineList = getRoutineIDs();

  if (routineList == nullptr) {

    errorPrint("No routines found");

    return;

  }

  int selectedRoutineID = -1;

  while (true) {

    clear();

    tft.println("Select Routine (* to go back):");

    int* ids = routineList->getIds();

    int count = routineList->getCount();

    for (int i = 0; i < count; i++) {

      tft.println(String(i + 1) + ". " + String(ids[i]));

    }

    char userSelection = promptSingle();

    if (userSelection == '*') {

      delete routineList;

      return;

    }

    int selectedRoutineIndex = userSelection - '0' - 1;

    if (selectedRoutineIndex >= 0 && selectedRoutineIndex < count) {

      selectedRoutineID = ids[selectedRoutineIndex];

      break;

    }

  }

  // Step 2: Show program details and confirm

  while (true) {

    Routine* routine = getRoutine(selectedRoutineID);

    if (routine == nullptr) {

      errorPrint("Routine not found");

      return;

    }

    clear();

    tft.println("Routine " + String(selectedRoutineID) + ":");

    int* durations = routine->getDurations();

    int* concentrations = routine->getConcentrations();

    int size = routine->getSize();

    for (int i = 0; i < size; ++i) {

      tft.println(String(i + 1) + ". Duration: " + String(durations[i]) + "m, Concentration: " + String(concentrations[i]) + "%");

    }

    tft.println("* to go back, # to continue");

    char userSelection = promptSingle();

    if (userSelection == '*') {

      delete routine;

      continue;  // Go back to program selection

    } else if (userSelection == '#') {

      delete routine;

      break;  // Proceed with the routine

    }

  }

  // REPAIRREPAIRREPAIRREPAIRREPAIRREPAIRREPAIRREPAIRREPAIRREPAIRREPAIRREPAIRREPAIRREPAIRREPAIRREPAIRREPAIRREPAIRREPAIR

  // Step 3: Prepare the system

  clear();

  tft.println("Preparing system");

  digitalWrite(pipeOpen, HIGH);

  topServo.write(servoOpen);

  digitalWrite(waterPump, HIGH);

  digitalWrite(EtOH95Pump, HIGH);

  digitalWrite(EtOH100Pump, HIGH);

  delay(timeTubeDrain);

  digitalWrite(pipeOpen, LOW);

  digitalWrite(waterPump, LOW);

  digitalWrite(EtOH95Pump, LOW);

  digitalWrite(EtOH100Pump, LOW);

  digitalWrite(pipeClose, HIGH);

  delay(timePipeSwitch);

  digitalWrite(pipeClose, HIGH);

  topServo.write(servoClosed);

  clear();

  tft.println("Load sample with liquid into bottom chamber");

  tft.println("Any key to continue");

  char confirmation = promptSingle();

  // Step 4: Load concentrations and manage duration

  Routine* routine = getRoutine(selectedRoutineID);

  if (routine == nullptr) {

    errorPrint("Routine not found");

    return;

  }

  int* durations = routine->getDurations();

  int* concentrations = routine->getConcentrations();

  int size = routine->getSize();

  clear();

  tft.println("Filling first solution: " + String(concentrations[0]) + "%");

  fill(concentrations[0]);

  drainBot();

  for (int i = 0; i < size; ++i) {

    clear();

    tft.println("Running program " + String(selectedRoutineID));

    tft.println("Step " + String(i + 1) + "/" + String(size));

    tft.println("Concentration: " + String(concentrations[i]) + "%, Duration: " + String(durations[i]) + "m");

    if (i < size - 1) {

      tft.println("~ETA left in step: " + String(durations[i]) + "m");

      tft.println("~ETA left in program: " + String(durations[i] + calculateRemainingTime(durations, i + 1, size)) + "m");

    }

    drainTop();

    if (i+1 < size){

      fill(concentrations[i+1]);

    }

    unsigned long durationMillis = (durations[i] * 60000UL) - timeToFill;

    unsigned long startMillis = millis();

    while (millis() - startMillis < durationMillis) {

      unsigned long elapsedMillis = millis() - startMillis;

      if (elapsedMillis % 60000 < 1000) {  // Refresh every minute

        clear();

        tft.println("Running program " + String(selectedRoutineID));

        tft.println("Step " + String(i + 1) + "/" + String(size));

        tft.println("Concentration: " + String(concentrations[i]) + "%, Duration: " + String(durations[i]) + "m");

        if (i < size - 1) {

          unsigned long etaLeftInStep = (durationMillis - elapsedMillis) / 60000UL;

          unsigned long etaLeftInProgram = etaLeftInStep + calculateRemainingTime(durations, i + 1, size);

          tft.println("~ETA left in step: " + String(etaLeftInStep) + "m");

          tft.println("~ETA left in program: " + String(etaLeftInProgram) + "m");

        }

        // Ensure this code runs only once per minute

        delay(1000);  // Add a short delay to avoid running this code multiple times in the same second

      } else {

        // Add a delay to reduce CPU usage while waiting for the next check

        delay(100);  // Adjust the delay as needed

      }

    }

  }

  // Step 5: Completion screen

  clear();

  tft.println("Complete!");

  tft.println(String(size) + " steps in " + String(calculateTotalTime(durations, size)) + " mins.");

  tft.println("Press any key to continue");

  char userSelection = promptSingle();

  // Clean up dynamically allocated memory

  delete routine;

  delete routineList;

}

void newRoutine() {

  int routineID = captureNumber("Enter 3-digit Routine ID:");

  while (routineID >= 1000 || routineID < 100) {

    errorPrint("ID must be 3 digits");

    routineID = captureNumber("Enter 3-digit Routine ID:");

  }

  DynamicJsonDocument doc(1024);

  JsonArray durations = doc.createNestedArray("durations");

  JsonArray concentrations = doc.createNestedArray("concentrations");

  int step = 1;

  while (true) {

    int durationInput = captureNumber("Duration for step " + String(step) + ":");

    durations.add(durationInput);

    int concentrationInput = captureNumber("Concentration for step " + String(step) + ":");

    concentrations.add(concentrationInput);

    clear();

    tft.println("Finished? " + String(step) + " steps have been entered. Press # to submit and any other key to continue");

    char userSelection = promptSingle();

    if (userSelection == '#') {

      addRoutineToJson(routineID, durations, concentrations);

      return;

    }

    step += 1;

  }

}

void viewRoutines() {

  RoutineIDs* routineList = getRoutineIDs();

  if (routineList == nullptr) {

    errorPrint("No routines found");

    return;

  }

  while (true) {

    clear();

    tft.println("View Routine (* to go back):");

    int* ids = routineList->getIds();

    int count = routineList->getCount();

    for (int i = 0; i < count; i++) {

      tft.println(String(i + 1) + ". " + String(ids[i]));

    }

    char userSelection = promptSingle();

    if (userSelection == '*') {

      break;

    }

    // Convert userSelection to an integer to select the routine

    int selectedRoutineIndex = userSelection - '0' - 1;  // Convert char to int and adjust for zero indexing

    if (selectedRoutineIndex >= 0 && selectedRoutineIndex < count) {

      viewRoutine(ids[selectedRoutineIndex]);  // Call viewRoutine with the selected routine ID

    }

  }

  // Clean up the dynamically allocated memory

  delete routineList;

}

void viewRoutine(int routineID) {

  Routine* routine = getRoutine(routineID);

  clear();

  tft.println("Routine " + String(routineID) + ":");

  int* durations = routine->getDurations();

  int* concentrations = routine->getConcentrations();

  int size = routine->getSize();

  for (int i = 0; i < size; ++i) {

    tft.println(String(i + 1) + ". Duration: " + String(durations[i]) + ", Concentration: " + String(concentrations[i]) + "%");

  }

  tft.println("Press # to continue");

  while (true) {

    char userSelection = promptSingle();

    if (userSelection == '#') {

      break;

    }

  }

  delete routine;

}

void deleteRoutine() {

  // Get the list of routine IDs using the getRoutineIDs function

  RoutineIDs* routineList = getRoutineIDs();

  if (routineList == nullptr) {

    errorPrint("No routines found");

    return;

  }

  while (true) {

    // Clear the TFT screen

    clear();

    tft.println("Delete Routine (* to go back):");

    // Display Routine IDs

    int* ids = routineList->getIds();

    int count = routineList->getCount();

    for (int i = 0; i < count; i++) {

      tft.println(String(i + 1) + ". " + String(ids[i]));

    }

    // Wait for user input

    char userSelection = promptSingle();

    switch (userSelection) {

      case '*':

        // Exit the function if the user presses '*'

        delete routineList;  // Clean up dynamically allocated memory

        return;

      default:

        // Convert userSelection to an integer to select the routine

        int selectedRoutineIndex = userSelection - '0' - 1;  // Convert char to int and adjust for zero indexing

        if (selectedRoutineIndex >= 0 && selectedRoutineIndex < count) {

          // Confirm deletion

          tft.println("Are you sure you want to delete routine " + String(ids[selectedRoutineIndex]) + "? # for yes.");

          char confirmation = promptSingle();

          if (confirmation == '#') {

            deleteRoutineFromJson(ids[selectedRoutineIndex]);  // Call deleteRoutineFromJson with the selected routine ID

            successPrint("Routine deleted");

          }

        }

        break;

    }

  }

}

void flush() {

  clear();

  tft.println("Prepare to flush all tubes / glassware");

  tft.println("Remove all white tubes from liquid source");

  tft.println("Attatch waste container to bottom");

  tft.println("Press # to start flush and * to exit");

  char userSelection = promptSingle();

  switch (userSelection) {

    case '#':

      clear();

      tft.println("Press any key to stop flushing");

      digitalWrite(pipeOpen, HIGH);

      topServo.write(servoOpen);

      digitalWrite(waterPump, HIGH);

      digitalWrite(EtOH95Pump, HIGH);

      digitalWrite(EtOH100Pump, HIGH);

      char confirmation = promptSingle();

      topServo.write(servoClosed);

      digitalWrite(waterPump, LOW);

      digitalWrite(EtOH95Pump, LOW);

      digitalWrite(EtOH100Pump, LOW);

      digitalWrite(pipeOpen, LOW);

      digitalWrite(pipeClose, HIGH);

      delay(timePipeSwitch);

      digitalWrite(pipeClose, LOW);

      return;

    case '*':

      return;

    default:

      flush();

      return;

  }

}

void sanityCheck(){

  clear();

  tft.println("This is to make sure that all hardware is wired correctly");

  tft.println("If at any point the machine is not acting how the screen describes it to, turn it off and troubleshoot, or troubleshoot all at the end");

  tft.println("Press # to continue and * to exit");

  char confirmation = promptSingle();

  switch (confirmation) {

    case '#':

      break;

    case '*':

      return;

  }

  clear();

  tft.println("Did the top servo turn back and forth?");

  for (int i = 0; i < 3; i++) {

    topServo.write(servoOpen);

    delay(500);

    topServo.write(servoClosed);

    delay(500);

  }

  tft.println("If not, the servo is not receiving power, it is unplugged, or the servo is on the wrong pin");

  tft.println("Any key to continue");

  confirmation = promptSingle();

  clear();

  tft.println("Is the servo in the open position (can let water through)?");

  topServo.write(servoOpen);

  tft.println("Any key to continue");

  confirmation = promptSingle();

  clear();

  tft.println("Is the servo in the closed position (stops water)?");

  topServo.write(servoClosed);

  tft.println("If these are swapped, swap the values of servoOpen and servoClosed, or edit the variables if they're misaligned");

  tft.println("Any key to continue");

  confirmation = promptSingle();

  clear();

  tft.println("You should hear the pipe open and close 3 times");

  for (int i = 0; i < 3; i++) {

    digitalWrite(pipeOpen, HIGH);

    delay(1000);

    digitalWrite(pipeOpen, LOW);

    delay(200);

    digitalWrite(pipeClose, HIGH);

    delay(1000);

    digitalWrite(pipeOpen, LOW);

    delay(200);

  }

  tft.println("If you don't, pipeOpen and pipeClose may be defined on the wrong pins, the pipe is not recieving power, the relay is broken, or their shared ground is off");

  tft.println("Any key to continue");

  confirmation = promptSingle();

  clear();

  tft.println("Is the pipe open? (can let water through)?");

  digitalWrite(pipeOpen, HIGH);

  delay(10000);

  digitalWrite(pipeOpen, LOW);

  tft.println("Any key to continue");

  confirmation = promptSingle();

  clear();

  tft.println("Is the pipe closed? (can't let water through)?");

  digitalWrite(pipeClose, HIGH);

  delay(10000);

  digitalWrite(pipeClose, LOW);

  tft.println("If these are swapped, swap the pin values for pipeOpen and pipeClose");

  tft.println("Any key to continue");

  confirmation = promptSingle();

  clear();

  tft.println("Now I will test the pumps. Please label using sharpie 'water' on the left pump, 'EtOH95' on the middle pump, and 'EtOH100' on the right pump");

  tft.println("Running water pump (you can feel the pumps to see which one is running)");

  digitalWrite(waterPump, HIGH);

  tft.println("Any key to continue");

  confirmation = promptSingle();

  digitalWrite(waterPump, LOW);

  clear();

  tft.println("Running EtOH95 pump");

  digitalWrite(EtOH95Pump, HIGH);

  tft.println("Any key to continue");

  confirmation = promptSingle();

  digitalWrite(EtOH95Pump, LOW);

  clear();

  tft.println("Running EtOH100 pump");

  digitalWrite(EtOH100Pump, HIGH);

  tft.println("Any key to continue");

  confirmation = promptSingle();

  digitalWrite(EtOH100Pump, LOW);

  clear();

  tft.println("Hopefully all 3 turned on. If not, they may be unplugged, relay might be unplugged..");

  tft.println("If they are swapped, edit the pin assignments");

  tft.println("Any key to continue");

  confirmation = promptSingle();

  successPrint("Testing Complete!");

}

void calibrate(){

  int start = -1;

  long timeToFillNew = -1;

  int timeTopDrainNew = -1;

  int timeBotDrainNew = -1;

  int timePipeSwitchNew = -1;

  int timeTubeDrainNew = -1;

  clear();

  tft.println("This will calibrate 5 variables");

  tft.println("Any key to continue");

  char confirmation = promptSingle();

  clear();

  tft.println("Calibrating timePipeSwitch");

  tft.println("Resetting pipe");

  digitalWrite(pipeClose, HIGH);

  delay(10000);

  digitalWrite(pipeClose, LOW);

  tft.println("When you press start, you will hear the pipe move. Press any key again once the pipe stops making sound");

  tft.println("Any key to start");

  confirmation = promptSingle();

  start = millis();

  digitalWrite(pipeOpen, HIGH);

  confirmation = promptSingle();

  digitalWrite(pipeOpen, LOW);

  timePipeSwitchNew = millis() - start;

  successPrint("timePipeSwitch set to " + String(timePipeSwitchNew) +"ms");

  clear();

  tft.println("Calibrating timeTubeDrain");

  tft.println("Place the white water pump pipe into water, and blue water pipe into the top of the funnel");

  tft.println("When water starts dripping out, press any key");

  tft.println("Any key to start");

  confirmation = promptSingle();

  digitalWrite(waterPump, HIGH);

  digitalWrite(EtOH100Pump, HIGH);

  digitalWrite(EtOH95Pump, HIGH);

  start = millis();

  confirmation = promptSingle();

  digitalWrite(waterPump, LOW);

  digitalWrite(EtOH100Pump, LOW);

  digitalWrite(EtOH95Pump, LOW);

  timeTubeDrainNew = millis() - start;

  successPrint("timeTubeDrain set to " + String(timeTubeDrainNew) +"ms");

  clear();

  tft.println("Calibrating timeToFill");

  tft.println("We will now calibrate how long it takes to fill up the bottom container. KEEP IN MIND THAT LOADED TISSUES WILL DECREASE CONTAINER VOLUME.");

  tft.println("When bottom container is almost as full as you like it, press any key");

  tft.println("Any key to start");

  topServo.write(servoOpen);

  digitalWrite(pipeClose, HIGH);

  delay(timePipeSwitchNew);

  digitalWrite(pipeClose, LOW);

  confirmation = promptSingle();

  digitalWrite(waterPump, HIGH);

  digitalWrite(EtOH100Pump, HIGH);

  digitalWrite(EtOH95Pump, HIGH);

  long startTimeToFill = millis();

  confirmation = promptSingle();

  digitalWrite(waterPump, LOW);

  digitalWrite(EtOH100Pump, LOW);

  digitalWrite(EtOH95Pump, LOW);

  timeToFillNew = (millis() - startTimeToFill) * 3;

  debugPrint("timeToFill set to " + String(timeToFillNew) +"ms");

  clear();

  tft.println("Calibrating timeBotDrain");

  tft.println("We will be calibrating how long the bottom container takes to drain. This value may almost be 0 due to how long timePipeSwitch is");

  tft.println("When all liquid is gone frome bottom comtainer, press any key");

  tft.println("Any key to start");

  confirmation = promptSingle();

  digitalWrite(pipeOpen, HIGH);

  delay(timePipeSwitchNew);

  digitalWrite(pipeOpen, LOW);

  start = millis();

  confirmation = promptSingle();

  timeBotDrainNew = millis() - start;

  successPrint("timeBotDrain set to " + String(timeBotDrainNew) +"ms");

  clear();

  tft.println("Calibrating timeTopDrain");

  tft.println("Loading top compartment with water");

  topServo.write(servoClosed);

  digitalWrite(waterPump, HIGH);

  digitalWrite(EtOH100Pump, HIGH);

  digitalWrite(EtOH95Pump, HIGH);

  delay(timeToFillNew/3);

  digitalWrite(waterPump, LOW);

  digitalWrite(EtOH100Pump, LOW);

  digitalWrite(EtOH95Pump, LOW);

  tft.println("When all liquid has drained from top comtainer, press any key");

  tft.println("Any key to start");

  confirmation = promptSingle();

  start = millis();

  topServo.write(servoOpen);

  confirmation = promptSingle();

  topServo.write(servoClosed);

  timeTopDrainNew = millis() - start;

  successPrint("timeTopDrain set to " + String(timeTopDrainNew) +"ms");

  updateCalibrationJson(timeToFillNew, timeTopDrainNew, timeBotDrainNew, timePipeSwitchNew, timeTubeDrainNew);

  tft.println("Variables all set! Please reboot system imediately to activate new calibration variables.");

  tft.println("Any key to continue");

  confirmation = promptSingle();

}

void viewCalibration() {

    DynamicJsonDocument doc(4096);

  if (!readJsonFromFile("ROUTINES.TXT", doc)) return nullptr;

    JsonObject calibration = doc["calibration"];

    long timeToFill = calibration["timeToFill"];

    int timeTopDrain = calibration["timeTopDrain"];

    int timeBotDrain = calibration["timeBotDrain"];

    int timePipeSwitch = calibration["timePipeSwitch"];

    int timeTubeDrain = calibration["timeTubeDrain"];

    clear();

    tft.println("Calibration Variables:");

    tft.println("Time to Fill: " + String(timeToFill) + " ms");

    tft.println("Time to Drain Top: " + String(timeTopDrain) + " ms");

    tft.println("Time to Drain Bottom: " + String(timeBotDrain) + " ms");

    tft.println("Time to Switch Pipe: " + String(timePipeSwitch) + " ms");

    tft.println("Time to Drain Tube: " + String(timeTubeDrain) + " ms");

    tft.println("* to return");

    // Wait for the user to press '*'

    while (true) {

        char userSelection = promptSingle();

        if (userSelection == '*') {

            return;

        }

        delay(100);  // Small delay to prevent excessive CPU usage

    }

}

**Data used to build the plot shown in Fig. 2**

| **Time (s)** | **3 replicates of pumped volume (ml)** | | |
| --- | --- | --- | --- |
| 3 | 3 | 3.1 | 3.2 |
| 6 | 6.5 | 6.6 | 6.4 |
| 12 | 12.5 | 12 | 12.4 |
| 40 | 42.5 | 42 | 43 |
| 80 | 88 | 88.5 | 88.4 |
| 90 | 100 | 101 | 102 |
| 120 | 135 | 132 | 133 |
| 180 | 200 | 205 | 203 |

**Data used to build the bar plots shown in Fig. 4f**

| **Condition** | **3 replicates of SNR (a.u.)** | | |
| --- | --- | --- | --- |
| Manual | 76 | 78 | 75 |
| Tissue processor | 79 | 81 | 80 |

**Data points extracted from image intensity profiles used for the analyses shown in Fig. 4c**

| **Distance (µm)** | **Intensity (a.u.)** |
| --- | --- |
| 0 | 1.8 |
| 2 | 2.1 |
| 4 | 1.9 |
| 6 | 2.2 |
| 8 | 1.7 |
| 10 | 2 |
| 12 | 2.3 |
| 14 | 1.8 |
| 16 | 2.1 |
| 18 | 1.9 |
| 20 | 2 |
| 22 | 2.2 |
| 24 | 1.9 |
| 26 | 2.3 |
| 28 | 3 |
| 30 | 4.5 |
| 32 | 6.8 |
| 34 | 11.5 |
| 36 | 19.8 |
| 38 | 32.4 |
| 40 | 47.9 |
| 42 | 61.5 |
| 44 | 72.8 |
| 46 | 78.4 |
| 48 | 75.9 |
| 50 | 79.1 |
| 52 | 74.6 |
| 54 | 60.8 |
| 56 | 43.1 |
| 58 | 26.2 |
| 60 | 13.4 |
| 62 | 6.9 |
| 64 | 3.4 |
| 66 | 2.5 |
| 68 | 2.1 |
| 70 | 1.8 |
| 72 | 2.2 |
| 74 | 1.9 |
| 76 | 2 |
| 78 | 2.3 |
| 80 | 1.7 |
| 82 | 2.1 |
| 84 | 2.4 |
| 86 | 1.9 |
| 88 | 2.2 |
| 90 | 1.8 |
| 92 | 2 |
| 94 | 2.3 |
| 96 | 1.9 |
| 98 | 2.1 |
| 100 | 1.8 |
| 102 | 2.2 |
| 104 | 1.9 |
| 106 | 2.3 |
| 108 | 1.8 |
| 110 | 2 |
| 112 | 2.4 |
| 114 | 1.9 |
| 116 | 2.2 |
| 118 | 1.8 |
| 120 | 2 |
| 122 | 2.3 |
| 124 | 1.9 |
| 126 | 2.1 |
| 128 | 1.7 |
| 130 | 2.2 |
| 132 | 1.9 |
| 134 | 2.3 |
| 136 | 1.8 |
| 138 | 2.1 |
| 140 | 1.9 |
| 142 | 2.4 |
| 144 | 1.8 |
| 146 | 2 |
| 148 | 2.3 |
| 150 | 1.9 |
| 152 | 2.2 |
| 154 | 2.6 |
| 156 | 3.9 |
| 158 | 7.2 |
| 160 | 12.8 |
| 162 | 22.5 |
| 164 | 37.9 |
| 166 | 54.1 |
| 168 | 66.8 |
| 170 | 74.5 |
| 172 | 78.9 |
| 174 | 81.2 |
| 176 | 79.4 |
| 178 | 80.6 |
| 180 | 78.8 |
| 182 | 71.9 |
| 184 | 59.3 |
| 186 | 43.5 |
| 188 | 26.8 |
| 190 | 13.9 |
| 192 | 7.1 |
| 194 | 3.6 |
| 196 | 2.4 |
| 198 | 1.9 |
| 200 | 2.1 |
| 202 | 1.8 |
| 204 | 2.3 |
| 206 | 1.9 |
| 208 | 2.2 |
| 210 | 1.8 |
| 212 | 2 |
| 214 | 2.4 |
| 216 | 1.9 |
| 218 | 2.1 |
| 220 | 1.8 |
| 222 | 2.2 |
| 224 | 1.9 |
| 226 | 2.3 |
| 228 | 1.8 |
| 230 | 2 |
| 232 | 2.4 |
| 234 | 1.9 |
| 236 | 2.2 |
| 238 | 1.8 |
| 240 | 2.1 |
| 242 | 1.9 |
| 244 | 2.3 |
| 246 | 1.8 |
| 248 | 2.1 |
| 250 | 1.9 |

**Data points extracted from image intensity profiles used for the analyses shown in Fig. 4c**

| **Distance (µm)** | **Intensity (a.u.)** |
| --- | --- |
| 0 | 2.1 |
| 2 | 1.9 |
| 4 | 2.3 |
| 6 | 1.8 |
| 8 | 2.2 |
| 10 | 1.9 |
| 12 | 2.4 |
| 14 | 2 |
| 16 | 1.8 |
| 18 | 2.1 |
| 20 | 1.9 |
| 22 | 2.3 |
| 24 | 1.8 |
| 26 | 2.2 |
| 28 | 3.1 |
| 30 | 4.7 |
| 32 | 7.6 |
| 34 | 12.8 |
| 36 | 21.4 |
| 38 | 34.6 |
| 40 | 49.8 |
| 42 | 66 |
| 44 | 80.4 |
| 46 | 74.6 |
| 48 | 63.2 |
| 50 | 44.8 |
| 52 | 27.5 |
| 54 | 14.2 |
| 56 | 7.6 |
| 58 | 3.8 |
| 60 | 2.6 |
| 62 | 2.1 |
| 64 | 1.9 |
| 66 | 2.3 |
| 68 | 1.8 |
| 70 | 2.2 |
| 72 | 1.9 |
| 74 | 2.4 |
| 76 | 1.8 |
| 78 | 2.1 |
| 80 | 1.9 |
| 82 | 2.3 |
| 84 | 1.8 |
| 86 | 2.2 |
| 88 | 1.9 |
| 90 | 2.1 |
| 92 | 1.8 |
| 94 | 2.4 |
| 96 | 1.9 |
| 98 | 2.2 |
| 100 | 1.8 |
| 102 | 2.3 |
| 104 | 1.9 |
| 106 | 2.1 |
| 108 | 1.8 |
| 110 | 2.4 |
| 112 | 1.9 |
| 114 | 2.2 |
| 116 | 1.8 |
| 118 | 2.1 |
| 120 | 2.4 |
| 122 | 1.9 |
| 124 | 2.2 |
| 126 | 1.8 |
| 128 | 2.1 |
| 130 | 2.3 |
| 132 | 1.9 |
| 134 | 2.2 |
| 136 | 1.8 |
| 138 | 2.4 |
| 140 | 1.9 |
| 142 | 2.2 |
| 144 | 1.8 |
| 146 | 2.1 |
| 148 | 2.4 |
| 150 | 2 |
| 152 | 2.6 |
| 154 | 3.8 |
| 156 | 6.9 |
| 158 | 12.4 |
| 160 | 22.8 |
| 162 | 38.6 |
| 164 | 55.9 |
| 166 | 69.8 |
| 168 | 77.6 |
| 170 | 81.2 |
| 172 | 83 |
| 174 | 81 |
| 176 | 82.4 |
| 178 | 80.2 |
| 180 | 78.5 |
| 182 | 72.1 |
| 184 | 59.6 |
| 186 | 44 |
| 188 | 27.9 |
| 190 | 14.6 |
| 192 | 7.5 |
| 194 | 3.7 |
| 196 | 2.4 |
| 198 | 1.9 |
| 200 | 2.2 |
| 202 | 1.8 |
| 204 | 2.3 |
| 206 | 1.9 |
| 208 | 2.2 |
| 210 | 1.8 |
| 212 | 2.1 |
| 214 | 2.4 |
| 216 | 1.9 |
| 218 | 2.2 |
| 220 | 1.8 |
| 222 | 2.3 |
| 224 | 1.9 |
| 226 | 2.1 |
| 228 | 1.8 |
| 230 | 2.4 |
| 232 | 1.9 |
| 234 | 2.2 |
| 236 | 1.8 |
| 238 | 2.1 |
| 240 | 2.3 |
| 242 | 1.9 |
| 244 | 2.2 |
| 246 | 1.8 |
| 248 | 2.1 |
| 250 | 1.9 |
