# Supplementary material for: Design and evaluation of low-cost, DIY programmable tissue processor for solvent exchange in biological sample preparation
Source: PLoS One. 2026 Mar 3;21(3):e0341033. doi: 10.1371/journal.pone.0341033 (PMC12956129; doi:10.1371/journal.pone.0341033)
Supplement: Attachment — Submitted filename: Comments to the authors.docx [file pone.0341033.s003.docx]

**Comments to the authors:**

**Title: “Low-cost, DIY programmable tissue processor for solvent exchange in biological sample preparation”**

**Manuscript Number**: **(PONE-D-25-48079)**

**Dear the editor of PLOS ONE Journal**

**I** would like to express my sincere gratitude for the opportunity to review this manuscript and contribute my expertise to the advancement of scientific knowledge. I also wish to extend my appreciation to the authors for their valuable contribution in exploring an innovative and time-efficient technology for tissue processing. Their work addresses a significant need in histological research and diagnostics by proposing a method that has the potential to reduce the time, labor, and resource demands traditionally associated with conventional tissue processing procedures.

1. **Title: “Low-cost, DIY programmable tissue processor for solvent exchange in biological sample preparation”** (PONE-D-25-48079):

- **Comment:** The title is concise, descriptive, and captures the core innovation of the work “low-cost,” “DIY,” and “programmable” are strong keywords that attract interest in open-source hardware and biological sample preparation.
- **Suggestions:**
- Consider specifying the type of tissues or applications (e.g., “for histological and fluorescence microscopy sample preparation”) to make the scope clearer.
- Describe the “DIY” first and make formal contexts since journals like *PLOS ONE* prefer academic tone.

### 2. ****Abstract****

- **Comment:** The abstract clearly summarizes the motivation, approach, design, and findings. It effectively emphasizes accessibility and cost reduction.
- **Suggestions:**
- The problem statement could briefly include **quantitative efficiency** (e.g., percentage reduction in manual time).
- Add a **brief mention of validation results** (e.g., comparable fluorescence intensity to manual staining).

3. **Introduction**

- **Comment:** The introduction is well structured, providing a logical flow from biological imaging needs to limitations of existing systems and the motivation for a low-cost alternative. References (1–16) are appropriate and current.
- **Suggestions:**
- Include a comparative statement with similar open-source automation tools (e.g., OpenTrons or other microfluidic DIY devices).
- Add a clear hypothesis or research question at the end of the section (e.g., “We hypothesize that a low-cost programmable tissue processor can achieve staining results equivalent to commercial systems”).
- Clarify the novelty, does the processor uniquely automate both dehydration and rehydration steps or simply adapt existing robotics principles?
- Check citation consistency (e.g., space before parentheses: “subsequent tissue clearing(6)” → “subsequent tissue clearing (6)”).

4. **Design of the Automated Tissue Processor**

- **Comment:** This section provides excellent technical clarity. Figures 1–2 support understanding.
- **Suggestions:**
- Include a brief cost breakdown within this section, not only in Table 1, to strengthen the claim of affordability.
- A risk assessment or safety note (e.g., ethanol vapor management) could improve completeness.
- Clarify scalability, can this handle multiple samples simultaneously?
- Add comparison with existing low-cost automation platforms for context (e.g., Arduino-controlled microfluidic systems).

5. **Software Design and Operation**

- **Comment:** Comprehensive description of the user interface and operational logic. The inclusion of a flowchart (Fig. 3) is excellent.
- **Suggestions:**
- Indicate whether the software includes error handling or alerts (e.g., empty solvent reservoirs).
- Consider including code versioning or repository DOI earlier for transparency.
- A short subsection on usability testing (who tested it, user feedback) could strengthen reproducibility claims.

6. **Performance of the Tissue Processor**

- **Comment:** Validation using propidium iodide (PI) staining on multiple plant species is appropriate and convincing. Quantitative fluorescence comparison (signal-to-noise ratio ~80) supports equivalency.
- **Suggestions:**
- Include statistical analysis (e.g., t-test comparing mean intensity between manual and automated samples).
- Provide replicate numbers (n) for reproducibility.
- Add discussion on long-term performance or maintenance stability (e.g., after repeated use).
- Minor: specify microscope model consistently (e.g., “Zeiss LSM 880 confocal microscope” should appear consistently, not “Zeiss u880”).

7. **Design Considerations**

- **Comment:** Good rationale for material and component selection.
- **Suggestions:**
- Add a table of solvent compatibility for quick reference.
- Include expected lifespan or maintenance recommendations for pumps and valves.
- Clarify whether temperature or humidity control is needed for reliable operation.
- Mention possible future modifications (e.g., incorporation of sensors or remote data logging).

8. **Conclusion**

- **Comment:** The conclusion effectively summarizes the study’s achievements and potential applications.
- **Suggestions:**
- Add a quantitative summary (e.g., cost reduction, processing time, and staining reproducibility).
- End with a forward-looking statement about potential expansion to animal tissues or integration with automated imaging systems. Example: “Future work could extend this platform to integrate real-time imaging or temperature-controlled sample handling.”

9. **Materials and Methods**

- **Comment:** Extremely detailed and well written, especially for reproducibility. The use of open-source files and a GitHub repository aligns with *PLOS ONE*’s data policy.
- **Suggestions:**
- For clarity, include subheadings under “Assembly Procedure” (e.g., “Control Box,” “Pump Module”).
- Add a short note on ethical or biosafety considerations if using plant materials.
- Ensure units and symbols follow consistent formatting (e.g., “°C,” “μm”).
- In “Electrical wiring,” specify any safety isolation between power and control circuits.
- Table 1 could include total estimated cost and country availability of parts.

10. **Figures and Tables**

- **Comment:** Figures are clear and informative. Table 1–3 provide high transparency.
- **Suggestions:**
- Ensure all figures include scale bars for microscopy images.
- Figures 1 and 7: add legends with abbreviations for clarity.
- Combine Fig. 2 (pump calibration) and a small chart comparing manual vs. automated results for visual impact.
- Verify all figure numbers are cited in the text sequentially.

11. **References**

- **Comment:** The reference list is recent, relevant, and includes both foundational and contemporary studies (e.g., PLOS ONE 2024 papers).
- **Suggestions:**
- Ensure reference consistency (some journal names italicized, others not).
- Add DOIs for all references where possible.
- A few references (e.g., “Tissue Processing Systems Market...”) are non-peer-reviewed and should be supported by primary sources if available. The status of the paper is ongoing.
- Include one or two recent citations (2023–2025) on open-source lab automation to contextualize innovation.

**Overall Assessment**

- **Strengths:**
- High reproducibility and transparency (open-source design, detailed methods).
- Excellent integration of engineering and biological applications.
- Figures and methods are well structured and clear.
- **Weaknesses & Improvements:**
- Limited statistical validation and quantitative comparison.
- Some redundancy across sections (especially Abstract vs. Conclusion).
- “DIY” phrasing may reduce perceived technical rigor.
- Lack of discussion on safety, error handling, and scalability.
